# Exploring the functional implications of brain architecture and connectivity: a declarative language framework

**DOI:** 10.1186/1471-2202-13-S1-P151

**Published:** 2012-07-16

**Authors:** Ivan Raikov, Mario Negrello, Thomas G Close, Shyam Kumar, Erik De Schutter

**Affiliations:** 1 Computational Neuroscience Unit, Okinawa Institute of Science and Technology, Okinawa, Japan; 2 University of Antwerp, Antwerp, Belgium

## 

**Figure 1:**
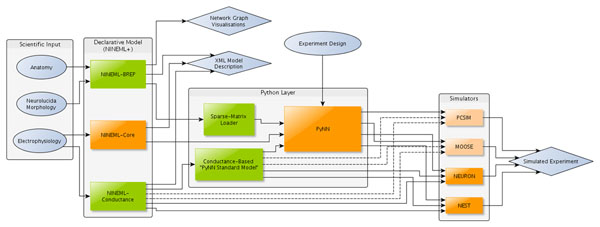


We present a prototype framework for exploring hypotheses about the neuroanatomical structures and connectivity in the cerebellar cortex at various levels of granularity, based on experimental data and hypotheses from the scientific literature [1]. As illustrated in Figure 1, the framework consists of declarative and algorithmic components. The declarative components include languages for describing connectivity and neuronal and synaptic mechanisms, built as an extension to the NineML description language [2]. The algorithmic components are Python scripts and the PyNN program, which are used for interfacing to specific simulator platforms and for simulation control.

The core assumptions of the framework are: 1) connectivity rules are specified as probability distributions for overlapping volumes of objects of different categories; 2) synapse locations are randomly generated from the distribution associated with an overlapping volume 3) the volumes that represent dendritic trees have regions of uniform synaptic density.

We use the NineML language for declarative descriptions of integrate-and-fire neuronal dynamics, and we have built two extensions to NineML to describe conductance-based neuronal spiking mechanisms and geometric connectivity. The NineML Conductance language is an extension of NineML for describing Ohmic and GHK currents based on the Hodgkin-Huxley formalisms or Markov chains. The NineML BREP language is an extension of NineML for constructive 3D boundary representation [3] of neuroanatomical structures and connectivity at various levels of granularity (from coarse-resolution solids to fine meshes). NineML BREP is implemented on top of the GNU Triangulated Surface library [4], and provides the ability to specify geometric parameters for the instantiation of topological objects, such as coordinates for placement, or probability distributions for random placement of a group of identical objects; define categories of topological objects, such as stellate, basket and Golgi cells; define rules for connectivity between different categories of objects.
